# Predictors of Long-Term Desensitization in Children Treated with Oral Immunotherapy for Food Allergy: A Real-World Cohort Study

**DOI:** 10.3390/jcm14134727

**Published:** 2025-07-03

**Authors:** Miyuki Hoshi-Funakawa, Mizuho Nagao, Norio Kawamoto, Hidenori Ohnishi, Takao Fujisawa

**Affiliations:** 1 Allergy Center, NHO Mie National Hospital, Tsu 514-0125, Japan; deep_snow_star@yahoo.co.jp (M.H.-F.); watersail711@gmail.com (M.N.); 2 Department of Pediatrics, Gifu University Graduate School of Medicine, Gifu 501-1194, Japan; ohnishi.hidenori.f1@f.gifu-u.ac.jp

**Keywords:** food allergy, oral immunotherapy, desensitization, full dose, oral discomfort, adverse events

## Abstract

**Background**: The degree of desensitization achieved through oral immunotherapy (OIT) varies widely. This study aimed to identify factors predictive of full desensitization—defined as daily, symptom-free consumption of allergenic foods—in children with food allergies under real-world conditions. **Methods**: A follow-up survey was conducted for all children who received OIT at Mie National Hospital, Japan, between 2008 and 2017, to assess long-term safety. Patients treated for hen’s egg, cow’s milk, or wheat allergy with at least five years of follow-up were included in the analysis. Data were collected from surveys and medical records, including treatment-emergent adverse events (TEAEs), baseline allergen-specific IgE (sIgE), and daily intake of allergenic foods. TEAEs were evaluated using the World Allergy Organization grading system. Full desensitization was defined as the ability to consume a typical daily portion of allergenic food without symptoms. Predictive factors were identified by multivariate logistic regression. **Results**: A total of 111 patients (142 OIT courses: egg *n* = 72, milk *n* = 47, wheat *n* = 23) met inclusion criteria. The median age at OIT initiation was 6 years (IQR: 5–8). No TEAEs of grade 4 or grade 5 were reported. Overall, 30% of them achieved full desensitization: 32% in the egg group, 24% in the milk group, and 39% in the wheat group. Low baseline sIgE and absence of mild oral symptoms during build-up were identified as significant predictors. **Conclusions**: Mild oral symptoms may signal lower likelihood of success. Monitoring them may support individualized OIT.

## 1. Introduction

Food allergies affect approximately 5–10% of individuals in developed countries [1,2,3]. It was previously believed that most children with food allergies would outgrow their symptoms by school age. However, studies have shown that the development of tolerance is often delayed or incomplete, requiring patients to maintain dietary avoidance while remaining at risk for accidental anaphylaxis [4,5]. Current clinical management strategies, which emphasize strict avoidance of trigger foods and the availability of epinephrine for emergency use [3,6,7] are insufficient to fully protect patients or restore normal dietary habits. Therefore, there is a critical need for therapeutic approaches that not only prevent severe allergic reactions but also enable safe and unrestricted food intake.

Oral immunotherapy (OIT) has become increasingly recognized as a viable treatment for food allergies, offering the potential to improve quality of life [8,9,10]. OIT involves the gradual introduction of allergenic foods, starting with sub-threshold doses and progressing through a build-up phase until a maintenance dose is achieved [11]. Although oral immunotherapy (OIT) has successfully desensitized many patients and has induced sustained unresponsiveness (SU) [12] in some cases, the optimal duration and conditions required to achieve SU remain unclear [13,14,15]. Consequently, desensitization—defined as the ability to safely consume allergenic foods under continued treatment—is currently regarded as a more attainable and clinically meaningful goal [11].

Low-dose oral immunotherapy (OIT) protocols have attracted increasing attention due to their improved safety profile, with a reduced incidence of IgE-mediated allergic reactions and treatment-emergent adverse events (TEAEs) [10,16,17,18,19]. Nevertheless, some patients are able to tolerate full dietary amounts of allergenic foods without experiencing adverse symptoms following OIT [20,21]. This raises the question of whether low-dose OIT should be universally adopted or tailored to individual patient characteristics, particularly in relation to the goal of achieving full desensitization. Although this therapeutic goal is of significant clinical relevance, the patient- or treatment-related factors that predict such outcomes remain inadequately understood.

The present study aimed to identify clinical and immunological factors associated with full desensitization in children undergoing oral immunotherapy (OIT) in a real-world setting. In addition, both severe TEAEs and mild TEAEs—specifically oral irritation and discomfort, which have often been overlooked in previous studies—were evaluated to support the development of more personalized OIT strategies.

## 2. Methods

### 2.1. Study Design and Participants

This study originated as a safety investigation conducted in response to an alert issued by the Japanese Society of Pediatric Allergy and Clinical Immunology, following a reported case of severe anaphylaxis with permanent sequelae during OIT in 2017 (https://www.jspaci.jp/news/member/20171114-464/ accessed on 3 July 2025).

The initial objective was to assess the safety profile of OIT practices at Mie National Hospital. Data collection and documentation were performed for internal quality assurance purposes, and no fatal or serious adverse events resulting in permanent sequelae were reported in the safety survey.

Building on this foundation, we performed a secondary analysis of an existing clinical dataset to explore clinical and immunological factors associated with full desensitization, as outlined in the study objectives. For consistency and clinical relevance, the analysis was limited to children who had undergone oral immunotherapy (OIT) for one of the three major food allergens in Japan—egg, milk, or wheat.

Eligible participants met the following criteria: (1) age 4 years or older at the initiation of OIT; (2) a documented history of food-induced anaphylaxis; (3) a positive result from a double-blind placebo-controlled oral food challenge (DBPCFC) with a reaction threshold of less than 900 mg of egg protein (approximately one-eighth of a whole heated hen’s egg), 396 mg of milk protein (approximately 12 mL of cow’s milk), or 312 mg of wheat protein (approximately 3.6 g of wheat bread); and (4) a positive allergen-specific IgE (sIgE) test for the corresponding food. Children with non-IgE-mediated food allergies, including eosinophilic gastrointestinal diseases or food protein-induced enterocolitis syndrome (FPIES), were excluded. To evaluate long-term outcomes, a follow-up survey of clinical status was conducted through 2022.

Informed consent was obtained from the legal guardians of all participants. The study protocol was reviewed and approved by the Ethics Committee of Mie National Hospital (approval number: 2021-106, 18 February 2022).

### 2.2. Oral Immunotherapy Protocol

Oral immunotherapy (OIT) was administered in two phases: an inpatient build-up phase lasting 3–4 weeks to establish an individualized maintenance dose, followed by a home-based maintenance phase. The initial dose was set at one-tenth of the threshold dose determined by the baseline double-blind placebo-controlled oral food challenge (DBPCFC). On the first day of the build-up phase, prophylactic antihistamines and leukotriene receptor antagonists were administered prior to dosing. If only mild local symptoms, such as perioral erythema or mild oral discomfort occurred after dosing, the subsequent dose (1.2 times the previous amount) was administered on the same day and subsequently continued twice daily. In contrast, if moderate to severe symptoms developed, the scheduled dose escalation was withheld, and a reduced dose was administered on the following day. The dosing was progressively increased during the build-up phase until either the symptom threshold was reached—defined as the occurrence of symptoms on two or three consecutive administrations—or the child successfully consumed the full target dose, corresponding to one whole cooked egg, 200 mL of milk, or one slice of wheat bread.

After determining each patient’s individualized maximum tolerated dose at rest, an exercise challenge was conducted to evaluate the risk of exercise-induced anaphylaxis. Based on the results, the final maintenance dose was adjusted to minimize the risk of post-exercise symptoms. Adjustments included either reducing the dose to half of the pre-exercise maximum or implementing strict post-dose exercise restrictions, depending on the individual patient’s needs and the preferences of the patient or their guardian.

During the maintenance phase, patients were instructed to consume the allergenic food dose daily. Further dose adjustments were allowed as needed to ensure the continued absence of symptoms.

Details of the oral immunotherapy protocol, including food preparation and protein quantification methods for each allergen, are provided in Supplementary Protocol S1.

### 2.3. Clinical Outcome

Clinical outcome data, based on patient- or guardian-reported responses, were obtained from the follow-up survey, as previously described. OIT outcomes were assessed according to the amount of allergenic food consumed with either no symptoms or only occasional mild, non-disruptive symptoms. To capture the full spectrum of desensitization, outcomes were categorized into four levels:

Level 1: Consumption of less than one-quarter of the full dose.

Level 2: Consumption of between one-quarter and the full dose.

Level 3: Consumption of the full dose with only mild symptoms—such as transient oral irritation—occurring occasionally but not consistently.

Level 4 (Full Desensitization): Consumption of the full dose without any symptoms, representing the practically optimal therapeutic goal [11].

### 2.4. Baseline Allergen-Specific IgE

Baseline levels of allergen-specific IgE (sIgE) to egg white, ovomucoid, milk, casein, wheat, and ω-5 gliadin were extracted from the hospital’s electronic medical records and used as candidate predictors in subsequent statistical analyses.

### 2.5. Treatment-Emergent Adverse Events (TEAE)

Immediate-type symptoms associated with OIT were recorded according to the World Allergy Organization (WAO) grading system [22], which classifies reactions into five severity grades (1 to 5), with grade 3 or higher defined as anaphylaxis. In addition, oral discomfort—such as irritation or itching during the consumption of allergenic foods—was specifically documented. Although such symptoms are not included in the WAO grading system, they are frequently observed during OIT. Based on our clinical experience, these symptoms tend to be less frequently reported by patients who ultimately achieve favorable outcomes. Therefore, we considered them potentially informative and included them as a specific variable in our analysis.

Non-immediate-type reactions, such as eosinophilic gastrointestinal disease (EGID), were documented separately.

### 2.6. Statistical Analysis

The prevalence of TEAEs was calculated for each food allergen and compared using Pearson’s χ^2^ test. Temporal trends in TEAE frequency across OIT phases (build-up, early maintenance, late maintenance) were evaluated using the Cochran–Armitage test for trend.

Clinical and treatment-related characteristics were compared between outcome groups (full vs. partial desensitization) using Pearson’s χ^2^ test for categorical variables and the Mann–Whitney U test for continuous variables. Sankey diagrams were generated using ChartExpo™ for Excel (PolyVista Inc., Houston, TX, USA) to visualize the temporal progression of desensitization outcomes.

A multivariate logistic regression analysis was performed to identify factors independently associated with full desensitization. Variables with a *p*-value < 0.1 in univariate analyses, as well as clinically relevant factors identified a priori (e.g., age, gender, comorbid allergic diseases), were included in the model. A decision tree analysis, using the same variables as the logistic model, was also performed to illustrate key predictors of desensitization. All statistical analyses were performed using JMP version 17 (SAS Institute Inc., Cary, NC, USA).

## 3. Results

### 3.1. Study Population

**Figure 1:**
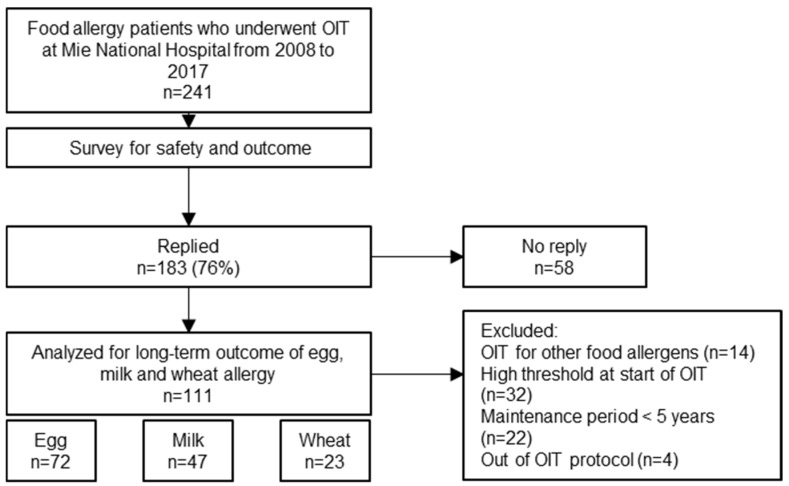
Flow diagram of patient selection for long-term outcome analysis.

Between 2008 and 2017, a total of 241 children with food allergies underwent oral immunotherapy (OIT) at our institution. Of these, 183 (76%) responded to the follow-up survey. After applying the predefined inclusion and exclusion criteria, 111 patients were included in the final analysis. These patients collectively underwent 142 OIT courses for egg (*n* = 72), milk (*n* = 47), and wheat (*n* = 23). Some patients received OIT for multiple allergens: two patients underwent OIT for all three foods; 21 underwent OIT for both egg and milk; four for egg and wheat; and two for milk and wheat (Figure 1). Compared with the 183 respondents, the 58 non-respondents did not differ significantly in age at OIT initiation or in the distribution of target allergens (egg, milk, and wheat) (Supplementary Table S1).

### 3.2. Participants’ Characteristics

The overall demographic and clinical characteristics of the study cohort were as follows. The median age at the start of oral immunotherapy (OIT) was 6 years (interquartile range [IQR]: 5–8), and the median current age was 15 years (IQR: 13–17). More than half of the participants (63.4%) were male. The median duration of the maintenance phase was 79 months (IQR: 66–89), reflecting long-term engagement with OIT. 

Table 1 presents the characteristics stratified by the target allergen (egg, milk, or wheat). Despite differences in the number of patients across allergen groups (egg: 72, milk: 47, wheat: 23), the median age, sex distribution, and duration of OIT were largely consistent across the groups. While the prevalence of comorbid bronchial asthma and atopic dermatitis appeared higher in the milk and wheat groups compared to the egg group, statistical analysis using the chi-square test revealed no significant differences among the groups. Baseline total and specific IgE levels were consistently high, indicating strong sensitization to the targeted allergens. The median DBPCFC thresholds for egg, milk, and wheat were equivalent to 1/46 of a whole egg, 2 mL of milk, and 1.2 g of wheat bread, respectively. Among patients who did not respond to the survey, baseline specific IgE levels for egg white and ovomucoid were significantly lower compared to respondents. Levels for wheat and ω-5 gliadin tended to be lower, though not significantly so, and median levels for milk and casein were also lower in non-respondents (Supplementary Table S1).

### 3.3. Safety

Table 2 presents treatment-emergent adverse events (TEAEs) observed during oral immunotherapy (OIT), stratified by allergen and treatment phase, and includes the results of Cochran–Armitage trend tests assessing changes over time. Importantly, no patients developed grade 4 or grade 5 symptoms at any phase of OIT across all allergen groups, indicating the absence of life-threatening reactions. Oral discomfort, such as mild irritation or itching during ingestion, was reported in approximately 30% of patients, with no significant differences among allergen groups without a clear trend of reduction over time. In contrast, mild TEAEs (WAO Grade 1/2) were most commonly observed during the build-up phase and generally showed a declining trend. Specifically, upper respiratory symptoms in the egg and milk groups, cutaneous symptoms in all groups, and gastrointestinal symptoms in the milk group showed statistically significant reductions. A numerical decline was also observed in gastrointestinal symptoms in the egg and wheat groups, and in upper respiratory symptoms in the wheat group, although these changes did not reach statistical significance.

Grade 3 symptoms, which are classified as anaphylaxis according to the WAO grading system, were observed primarily during the build-up phase in 6.9%, 17.0%, and 17.4% of patients receiving OIT for egg, milk, and wheat, respectively. During the early maintenance phase, 21.3% of milk OIT recipients experienced grade 3 symptoms, whereas the prevalence remained below 10% in the egg and wheat groups. In the late maintenance phase, grade 3 symptoms occurred in fewer than 10% of patients across all groups. Although a decreasing trend was observed over time, the change did not reach statistical significance, likely due to the overall low frequency of grade 3 events. Notably, none of the grade 3 events involved cardiovascular compromise or severe airflow limitation, which are typically associated with high-risk anaphylaxis.

Eosinophilic gastrointestinal disease (EGID) occurred in one patient receiving egg OIT and in two patients receiving milk OIT during the build-up phase, and in one additional milk OIT patient during the late maintenance phase. In all cases, OIT was discontinued following the diagnosis.

### 3.4. OIT Outcomes

**Figure 2:**
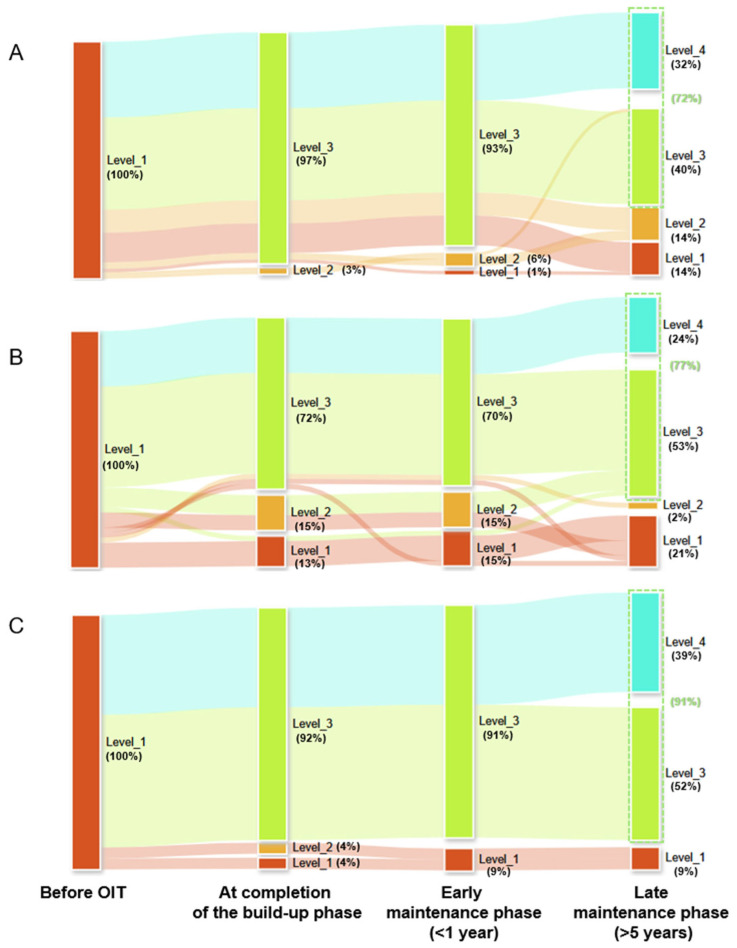
Longitudinal desensitization outcomes for each allergen group. Sankey diagrams illustrate the temporal progression of desensitization levels from the start of OIT through the build-up, early maintenance (<1 year), and late maintenance (>5 years) phases. (**A**) Egg OIT, (**B**) Milk OIT, (**C**) Wheat OIT. Each stream represents the proportion of patients at each desensitization level (described in Methods). Abbreviation: OIT, oral immunotherapy.

All patients were classified as level 1 at baseline. At the end of the build-up phase, 97%, 72%, and 92% of patients had reached level 3 for egg, milk, and wheat OIT, respectively (Figure 2). During the early maintenance phase, the proportion of patients with level 3 outcomes declined slightly to 93%, 70%, and 91% for egg, milk, and wheat, respectively.

At the end of the observation period (late maintenance phase), 72%, 77%, and 91% of patients had achieved level 3 or level 4 outcomes for egg, milk, and wheat, respectively. The proportion of patients achieving full desensitization (level 4) was 32% in the egg group, 24% in the milk group, and 39% in the wheat group.

### 3.5. Factors Associated with Full Desensitization

Among all patients, 43 out of 142 (30%) OIT courses resulted in full desensitization (Table 3). Median levels of allergen-specific IgE to milk, casein, and wheat were significantly lower in the full desensitization group compared to the non-full desensitization group (*p* = 0.005, 0.010, and 0.006, respectively). In contrast, sIgE levels to egg white, ovomucoid, and ω-5 gliadin did not differ significantly between the two groups.

The initial threshold dose of milk protein was significantly higher in the full desensitization group compared to the non-full desensitization group (median: 198 mg vs. 39.6 mg, *p* = 0.037), whereas no significant differences were observed in the initial threshold doses for egg or wheat.

Treatment-emergent adverse events (TEAEs) during the build-up phase were significantly less frequent in the full desensitization group. Specifically, oral discomfort (25.6% vs. 43.4%, *p* = 0.044), upper respiratory symptoms (27.9% vs. 51.5%, *p* = 0.009), cutaneous symptoms (48.8% vs. 66.7%, *p* = 0.045), and grade 3 events (2.3% vs. 16.2%, *p* = 0.020) were all significantly less common among patients who achieved full desensitization.

Other demographic and clinical characteristics—including age, sex, total IgE levels, and comorbid allergic diseases—did not differ significantly between the two groups.

A multivariate logistic regression analysis was performed to identify independent predictors of full desensitization. The model included age, gender, comorbid asthma and atopic dermatitis, type of allergenic food (egg, milk, or wheat), baseline allergen-specific IgE (sIgE) levels, threshold dose during the initial oral food challenge, and the occurrence of TEAEs during the build-up phase. To account for variability in baseline allergen-specific IgE (sIgE) levels among allergens, ROC analyses were performed to identify optimal cut-off values for predicting full desensitization. The area under the curve (AUC) was highest for wheat (0.841), followed by milk (0.775), and was modest for egg white (0.577). The optimal thresholds—determined using the Youden index—were 23.6 kUA/L for egg white, 14.9 kUA/L for milk, and 30.2 kUA/L for wheat (Supplementary Table S2). These values were used to dichotomize sIgE levels for inclusion in the multivariate analysis.

In the final model, two variables were significantly associated with full desensitization: sIgE levels below the cut-off (OR 4.14; 95% CI, 1.64–10.47; *p* = 0.003) and absence of oral discomfort during the build-up phase (OR 3.53; 95% CI, 1.32–9.47; *p* = 0.012). The absence of grade 3 symptoms also showed an elevated odds ratio (OR 8.07), though it did not reach statistical significance (*p* = 0.067) (Table 4).

**Figure 3:**
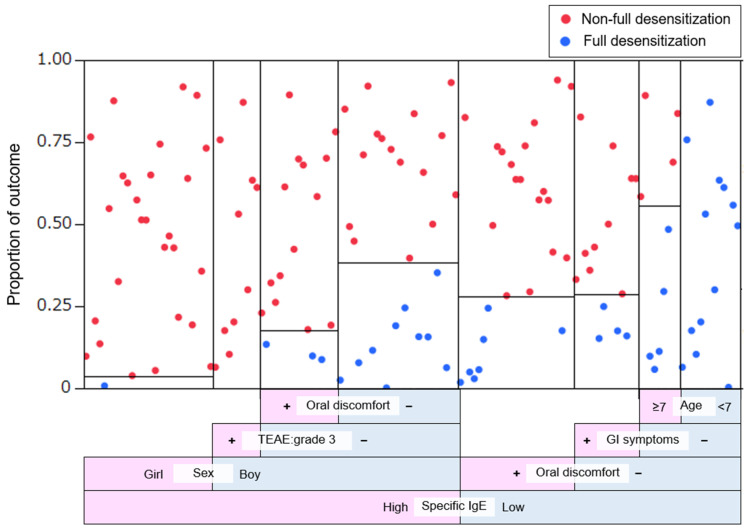
Decision tree model predicting full desensitization outcomes. The vertical axis indicates the proportion of patients achieving full (blue) and non-full (red) desensitization at each node of the decision tree. The horizontal axis represents the sequence of binary splits based on clinical and immunological predictors. A total of eight splits were included in the model, yielding an R^2^ value of 0.327. The area under the receiver operating characteristic (ROC) curve was 0.841. Predictive variables incorporated into the model included sex, baseline specific IgE level, oral discomfort, grade 3 treatment-emergent adverse events (TEAEs), gastrointestinal (GI) symptoms, and comorbid bronchial asthma (BA). “Age” refers to the patient’s age at the start of the OIT build-up phase.

In parallel, a decision tree analysis was conducted using the same variables to visualize the hierarchical structure of predictive factors (Figure 3). This analysis identified baseline sIgE level as the most informative predictor, followed by oral discomfort and sex. Specifically, among all patients (both boys and girls), those with high sIgE levels who also experienced grade 3 symptoms during the build-up phase had only a 2.5% probability of achieving full desensitization. In contrast, patients with low sIgE levels, no gastrointestinal or oral symptoms, and age under 7 years achieved full desensitization in 100% of cases.

## 4. Discussion

This study evaluated the long-term real-world outcomes of oral immunotherapy (OIT) in children with food allergies to three common allergens: egg, milk, and wheat. In addition, it identified clinical and immunological factors associated with favorable OIT outcomes. Given the lack of a universally accepted definition of sustained unresponsiveness (SU) [23] or “true tolerance” induced by OIT, we adopted a pragmatic outcome measure: the ability to consume a full daily dose of the allergenic food without symptoms in everyday life [11]. Our findings demonstrated that lower baseline allergen-specific IgE levels and the absence of oral discomfort during the build-up phase were significant predictors of successful desensitization.

First, we found that approximately one-third of OIT courses resulted in level 4 desensitization—defined as the ability to consume a full daily dose of the allergenic food without any symptoms—with success rates of 32% for egg, 24% for milk, and 39% for wheat. When including level 3 desensitization, defined as full-dose consumption with only occasional, mild, and non-disruptive symptoms, the rates increased substantially to 72%, 77%, and 81%, respectively. Importantly, no grade 4 or grade 5 TEAEs were observed throughout the OIT period. Although some patients experienced grade 3 reactions, their frequency was relatively low, and such events declined markedly during the late maintenance phase. These findings suggest that, under a carefully monitored and individualized protocol, OIT can be performed with an acceptable safety profile in pediatric patients. These findings are consistent with prior studies demonstrating the potential for desensitization and sustained unresponsiveness (SU) following OIT for egg, milk [24], and wheat allergies [25].

However, the achievement of OIT treatment goals is influenced by multiple factors, including the immunological heterogeneity of patients [26], individual preferences and goals [27] and socioeconomic context [28]. As a result, both the target quantity and frequency of allergenic food consumption during OIT can vary widely—from minimal maintenance doses aimed at preventing anaphylaxis due to accidental exposure, to unrestricted intake as part of a normal diet. In our protocol, the maintenance dose was individually tailored based on each patient’s tolerability, with some maintaining the full target dose and others continuing at a reduced dose. Given this personalized approach, we consider a full desensitization rate of approximately 30% to be clinically meaningful and achievable in real-world settings.

The diversity of treatment goals and patient profiles in OIT underscores the importance of shared decision-making (SDM) in clinical practice [29]. SDM involves a collaborative process in which physicians and families work together to select a treatment strategy that aligns with the patient’s clinical characteristics, risk tolerance, and lifestyle. In our study, some patients pursued full desensitization with unrestricted dietary intake, while others prioritized protection against accidental exposure, opting for lower maintenance doses. By allowing for individualized dose adjustment during the maintenance phase, our protocol reflects the principles of SDM and supports the integration of patient preferences into treatment planning.

Lower allergen-specific IgE (sIgE) levels are well-established predictors of favorable outcomes in oral immunotherapy (OIT) [30], and our findings are consistent with this observation. In addition to these established markers, our study revealed a novel clinical predictor: the presence of oral discomfort during the build-up phase. Although often regarded as a minor and self-limiting symptom, oral discomfort was identified as a negative predictor of full desensitization. This symptom may reflect local mast cell degranulation in the oral mucosa or activation of sensory neural pathways. Patients with food allergies frequently develop strong aversions to allergenic foods, which can serve as a barrier to the initiation and continuation of OIT [31]. In animal models, ovalbumin-sensitized mice have been shown to avoid allergen-containing sweetened solutions—normally preferred by non-sensitized controls [32,33]. This behavior was associated with elevated sIgE and interleukin-4 levels, yet did not elicit systemic allergic reactions. These findings suggest that food aversion may be mediated by neuroimmune interactions independent of overt anaphylaxis, potentially involving central nervous system processing. We hypothesize that similar neuroimmune mechanisms may underlie oral discomfort and food avoidance behavior in human OIT. If so, early aversive responses could impair desensitization success by promoting reduced adherence or heightened perception of allergenic stimuli. Further studies are warranted to elucidate the interplay between mucosal immune responses, sensory perception, and central modulation in the development of allergen tolerance.

This study has several limitations. First, it was an observational study conducted under real-world conditions; therefore, the results may not be directly comparable to those of randomized controlled trials. Nevertheless, real-world evidence is increasingly recognized as valuable, especially given the growing implementation of oral immunotherapy (OIT) in diverse clinical settings. Second, desensitization outcomes were assessed through a structured survey rather than a standardized oral food challenge (OFC). Although OFCs are widely regarded as the gold standard, they may not accurately reflect patients’ true clinical tolerance in daily life and may either overestimate or underestimate the actual ability to consume allergenic foods safely [34,35]. Third, sustained unresponsiveness (SU) was not included as an outcome. While SU is often considered a benchmark in clinical trials, it requires prolonged food avoidance, which may be impractical and potentially detrimental when managing staple foods such as egg, milk, and wheat. In fact, extended elimination following desensitization may reduce the likelihood of achieving long-term tolerance [36,37]. Fourth, although this study identified low baseline specific IgE levels and the absence of mild oral symptoms during the build-up phase as predictors of successful OIT, mechanistic insights remain limited due to the lack of immunological assessments such as basophil activation tests or epitope binding analyses. Fifth, the outcomes were based on long-term follow-up under real-world conditions, which may be subject to reporting bias. However, these endpoints also reflect clinically relevant, patient-centered measures that are crucial for decision-making in everyday practice. Finally, although not all patients responded to the follow-up survey, we examined the potential impact of this incomplete response. One concern is that non-respondents may have had poorer clinical outcomes and thus refrained from participating. However, our analysis showed that the non-respondent group had lower baseline specific IgE levels—particularly for egg white and ovomucoid (statistically significant), and for wheat and ω-5 gliadin (trend level). Since lower baseline specific IgE levels are generally associated with better treatment outcomes, it is plausible that some non-respondents may have experienced clinical remission and felt no need to participate in the survey. Taken together, this suggests that the results of the present study may be conservative, as patients with more favorable outcomes could have been underrepresented in the follow-up data

In conclusion, this study provides real-world evidence supporting both the safety and effectiveness of oral immunotherapy (OIT) for common pediatric food allergies and underscores the importance of individualized treatment strategies. The identification of low baseline specific IgE levels and the absence of early oral discomfort as predictors of full desensitization may aid in patient selection and risk stratification. These findings reinforce the need to align treatment goals with patient and family preferences through shared decision-making. Future research should further explore the immunologic and neuroimmune mechanisms underlying desensitization outcomes and aim to develop predictive biomarkers that can facilitate more personalized OIT approaches.

## Figures and Tables

**Table 1:** Characteristics of the subjects.

	Allergen Food for OIT		
	Egg	Milk	Wheat
Number of patients	72	47	23
Age (years) at start of OIT, median (IQR)	6 (5–9)	5 (5–7)	6 (5–8)
Current age (years); median (IQR)	15 (14–18)	14 (13–16)	14 (13–15)
Sex, male %	56	68	78
Treatment length (months); median (IQR)	81 (70–91)	80 (64–88)	73 (62–83)
Comorbid allergic diseases			
Bronchial asthma, %	42	64	65
Atopic dermatitis, %	38	62	57
Total IgE at baseline (IU/mL); median (IQR)	1107 (680–3097)	916 (605–1676)	1159 (596–1351)
Specific IgE, median (IQR) U_A_/L at baseline			
Egg white	24.7 (11.4–65.7)	-	-
Ovomucoid	13.5 (8.6–43.0)	-	-
Milk	-	30.2 (8.7–76.5)	-
Casein	-	26.5 (8.6–80)	-
Wheat	-	-	55.9 (21.5–133.7)
ω5-gliadin	-	-	4.39 (0.9–8.3)
DBPCFC threshold, mg protein, (IQR)			
Egg	156 (37.8–420)	-	-
Milk	-	66 (19.8–198)	-
Wheat	-	-	101 (41.9–163.9)

**Table 2:** Treatment-emergent adverse events (TEAEs) during oral immunotherapy (OIT) stratified by allergen and treatment phase.

TEAE	Treatment Phase	Egg, *n*, %		*p*-Value *	Milk, *n*, %		*p*-Value *	Wheat, *n*, %		*p*-Value *
Oral discomfort	Build-up	33	45.8%	n.s.	15	31.9%	n.s.	6	26.1%	n.s.
	Early maintenance	14	19.7%		11	23.4%		2	8.7%	
	Late maintenance	27	37.5%		17	36.2%		8	34.8%	
WAO Grading										
Grade 1 Upper respiratory	Build-up	23	32.0%	0.0397	29	61.7%	0.0004	11	47.8%	n.s.
	Early maintenance	24	33.3%		34	72.3%		9	39.1%	
	Late maintenance	12	16.7%		12	25.5%		6	26.1%	
Grade 1/2 Cutaneous	Build-up	38	52.8%	0.0025	35	74.5%	0.0001	14	60.9%	0.0388
	Early maintenance	35	48.6%		37	78.7%		12	52.2%	
	Late maintenance	20	27.8%		17	36.2%		7	30.4%	
Grade 1/2 Gastrointestinal	Build-up	34	47.2%	n.s.	21	44.7%	0.0200	7	30.4%	n.s.
	Early maintenance	28	38.9%		24	51.1%		6	26.1%	
	Late maintenance	24	33.3%		10	21.3%		6	26.1%	
Grade 3	Build-up	5	6.9%	n.s.	8	17.0%	n.s.	4	17.4%	0.0880
	Early maintenance	6	8.3%		10	21.3%		0	0.0%	
	Late maintenance	3	4.2%		3	6.4%		1	4.4%	
Grade 4/5	Build-up	0	-	-	0	-	-	0	-	-
	Early maintenance	0	-		0	-		0	-	
	Late maintenance	0	-		0	-		0	-	

* Cochran–Armitage test for trend. n.s.: not significant.

**Table 3:** Comparison of clinical characteristics and TEAEs between patients with and without full desensitization following OIT.

	Full Desensitization	Non-Full Desensitization	*p* Value ^#^
Number of patients	43	99	
Chicken egg/cow’s milk/wheat, *n*	23/11/9	49/36/14	0.368
Age at start of OIT (y); median (IQR)	6 (5–9)	6 (5–8)	0.404
Current age (y); median (IQR)	13 (11–17)	13 (11–14)	0.148
Gender (boy, %)	31 (72%)	59 (60%)	0.155
Comorbid allergic diseases, *n* (%)			
Asthma	25 (58%)	60 (61%)	0.815
Atopic dermatitis	24 (56%)	56 (57%)	0.899
Total IgE, median (IQR), IU/mL	977.5 (625.5–1670)	1107 (668–1832)	0.156
Specific IgE, median (IQR), kUA/L			
Egg white	20.3 (10.7–47.5)	28.3 (12.9–69.6)	0.214
Ovomucoid	11 (5.8–54.1)	16.2 (8.9–40.3)	0.377
Milk	8.8 (3.0–21.2)	43.9 (13.7–81.7)	0.005
Casein	8.1 (4.63–31.8)	51.2 (10.8–93.8)	0.010
Wheat	20.5 (7.63–27.7)	90.4 (37.5–171.5)	0.006
ω5-gliadin	1.4 (0.54–3.83)	5.5 (3.19–15.6)	0.309
Initial threshold, median (IQR), mg:protein			
Chicken egg	240 (54–522)	132 (36–360)	0.279
Milk	198 (82.5–264)	39.6 (19.8–173)	0.037
Wheat	101 (50.7–152)	77.7 (28.7–166)	0.466
TEAE in build-up phase, *n* (%)			
Oral discomfort	11 (25.6%)	43 (43.4%)	0.044
Grade 1_upper respiratory	12 (27.9%)	51 (51.5%)	0.009
Grade 1/2_cutaneous	21 (48.8%)	66 (66.7%)	0.045
Grade 1/2_gastrointestinal	14 (32.6%)	48 (48.5%)	0.079
Grade 3	1 (2.3%)	16 (16.2%)	0.020

^#^ Chi-square test was employed for categorical values and Man–Whitney U test was used for continuous valuables.

**Table 4:** Factors associated with full desensitization by multivariate logistic regression analysis.

Factors	OR	95% CI	*p* Value
Specific IgE < cut-off levels	4.14	1.64–10.47	0.003
Absence of oral discomfort during build-up phase	3.53	1.32–9.47	0.012
Absence of grade 3 symptoms during build-up phase	8.07	0.86–75.13	0.067

OR: odds ratio, CI: confidence interval.

## Data Availability

The data are in computers that are not connected to the Internet. Whenever disclosure is needed, the authors are ready to provide the information.

## Supplementary Materials

The following supporting information can be downloaded at: https://www.mdpi.com/article/10.3390/jcm14134727/s1, Protocol S1: Oral Immunotherapy Protocol for Egg, Milk, and Wheat Allergies; Figure S1: Overview of the Oral Immunotherapy Course: Build-up and Long-term Maintenance with Individualized Dose Adjustment.; Table S1: Comparison Between Respondents and Non-Respondents in the Follow-Up Survey of Oral Immunotherapy Patients.; Table S2: Receiver Operating Characteristic (ROC) Analysis of Allergen-Specific IgE Levels for Predicting Full Desensitization.

## Abbreviations

The following abbreviations are used in this manuscript:

TEAE	Treatment-Emergent Adverse Events
sIgE	Specific Immunoglobulin E
OIT	Oral Immunotherapy
DBPCFC	Double-Blind Placebo-Controlled Oral Food Challenge
EPIES	Food Protein-Induced Enterocolitis Syndrome
WAO	World Allergy Organization
