# Supplementary material for: Predictors of Long-Term Desensitization in Children Treated with Oral Immunotherapy for Food Allergy: A Real-World Cohort Study
Source: J Clin Med. 2025 Jul 3;14(13):4727. doi: 10.3390/jcm14134727 (PMC12251376; doi:10.3390/jcm14134727)
Supplement: Supplementary file 1 [file jcm-14-04727-s001.zip › jcm-3668959-supplementary.pdf]

Supplementary Protocol S1
Oral Immunotherapy Protocol for Egg, Milk, and Wheat Allergies

Eligibility Criteria for Inclusion in Analysis
Data from children were included in the present analysis if all of the following criteria were met:

(1) age ≥4 years,
(2) a documented history of food-induced anaphylaxis,
(3) a positive double-blind placebo-controlled oral food challenge (DBPCFC) with a threshold dose of less than 525 mg egg white protein (approximately 1/8 of a whole boiled hen's egg), 396 mg milk protein (approximately 12 mL cow's milk), or 312 mg wheat protein (approximately 3.6 g of wheat bread or 12 g of udon noodles),
(4) detectable serum specific IgE (sIgE) to the relevant allergen.

Protocol Overview

OIT was initiated with a double-blind placebo-controlled oral food challenge (DBPCFC) to determine the individual threshold dose for each patient. The therapy consisted of two phases: a build-up phase (3–4 weeks, in-hospital), and a maintenance phase (at-home).

On the first day of the build-up phase, patients ingested one-tenth of the threshold dose determined by DBPCFC. Pre-treatment with antihistamines and leukotriene receptor antagonists was routinely administered. Dose escalation occurred twice daily by increasing the dose to 1.2 times the previous amount if no or only mild local symptoms (e.g., perioral erythema, oral discomfort) were observed. In cases of moderate to severe reactions, dose escalation was halted and resumed at a reduced dose on the following day.

Build-up was terminated either upon repeated symptom induction (two to three consecutive episodes) or once the patient reached a predetermined target maintenance dose. The target was individualized to the highest dose tolerated without inducing symptoms and typically corresponded to a full serving (e.g., one whole cooked egg, 200 mL of milk, or a full staple portion of wheat). An exercise challenge was then performed to confirm the absence of exercise-induced symptoms, and the final maintenance dose was adjusted accordingly.

Patients continued daily ingestion at home and were monitored regularly. Dosage adjustments were permitted to maintain minimal or no symptoms during maintenance.

Preparation and Protein Quantification of Allergenic Foods

Due to the real-world nature of the study, we did not use standardized allergen extracts. Instead, food materials were selected and prepared to ensure consistent and reproducible protein dosing as follows:

Egg:

The primary form used was hard-boiled whole egg. However, for doses below 1 gram of egg white protein, it was difficult to accurately measure small quantities using cooked material. In these cases, we used whole-egg dry powder (containing equivalent protein amounts) mixed thoroughly into a food matrix such as pumpkin paste to achieve precise dosing.

Milk:

Commercial cow's milk was used as the standard source. For doses below 1 mL, we prepared a 1:10 dilution in water to allow for accurate volume measurement and protein dosing.

Wheat:

We primarily used boiled udon noodles because they can be easily divided and weighed, allowing for more accurate and reproducible measurement of protein content by weight. For doses under 1 gram of wheat protein, wheat flour powder was carefully mixed into pumpkin paste to enable accurate weighing and administration. As the dose increased, patients were allowed to choose their preferred food form; some received white bread portions matched for equivalent protein content instead of noodles during the maintenance phase.

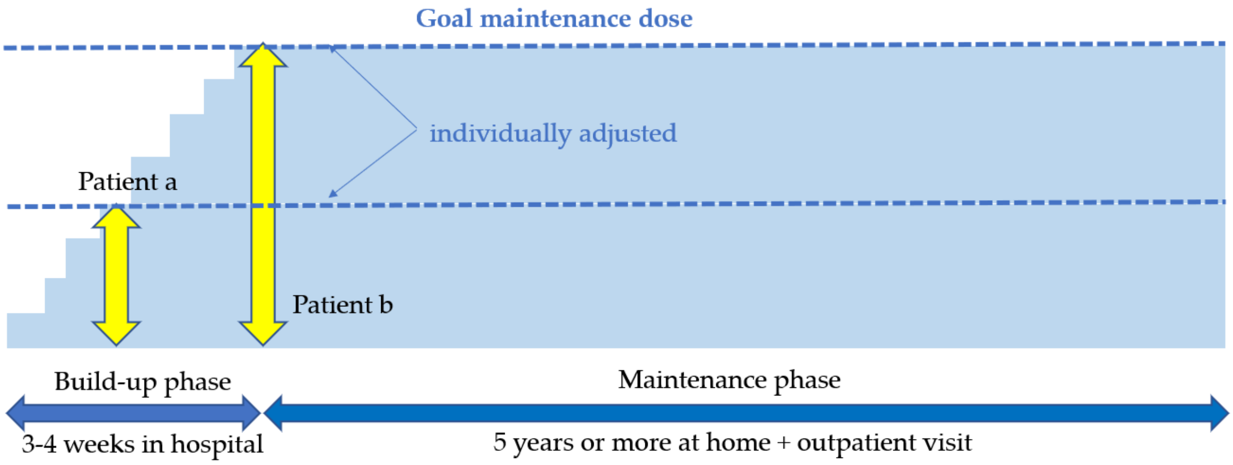

Figure S1 Overview of the Oral Immunotherapy Course: Build-up and Long-term Maintenance with Individualized Dose Adjustment

Table S1 Comparison Between Respondents and Non-Respondents in the Follow-Up Survey of Oral Immunotherapy Patients

	Response to the survey		
	Responded	Not responded	P value
Number of patients	183	58	
Median age at start of OIT (y)	8	10	0.504
Allergen food for OIT			
Egg	105	33	0.408
Milk	79	23	
Wheat	37	6	
Specific IgE, median, kUA/L			
Egg white	22.9	7.9	0.012
Ovomucoid	16.6	5.8	0.018
Milk	27.3	17.3	0.221
Casein	23.2	17.4	0.158
Wheat	45.3	16.7	0.095
ω5-gliadin	4.1	1.5	0.099

Table S2 Receiver Operating Characteristic (ROC) Analysis of Allergen-Specific IgE Levels for Predicting Full Desensitization

Allergen	AUC	Optimal cut-off (kUA/L)	Sensitivity	Specificity	P-value
Egg white	0.577	23.6	0.571	0.609	0.392
Milk	0.775	14.9	0.750	0.727	0.006
Wheat	0.841	30.2	0.857	0.778	0.021

For each allergen (egg white, milk, and wheat), receiver operating characteristic (ROC) analysis was performed to assess the ability of baseline allergen-specific IgE (sIgE) levels to discriminate between patients who achieved full desensitization and those who did not. The optimal cut-off value was determined by maximizing the Youden index (sensitivity + specificity – 1). The area under the ROC curve (AUC), optimal cut-off, sensitivity, specificity, and corresponding p-values were reported for each allergen.

Abbreviations: AUC, area under the ROC curve; ROC, receiver operating characteristic.

The optimal cut-off value for each allergen was calculated using the Youden index.

P-values were derived from the likelihood ratio test.
